# A Phase III, Randomized, Double-Blind, Active-Controlled Non-Inferiority Trial Evaluating the Immunogenicity and Safety of Gardisun, a Quadrivalent Human Papillomavirus Vaccine, Compared with Gardasil^®^ in Healthy Volunteers Aged 15–35 Years

**DOI:** 10.3390/vaccines14060540

**Published:** 2026-06-18

**Authors:** Erfan Pakatchian, Minoo Mohraz, Mohammad Taghavian, Babak Javadimehr, Hajar Mohammadi Barzelighi, Majid Teymoori-Rad, Mehrdad Ghodsi, Zahra Naderi Saffar

**Affiliations:** 1 Biosunpharmed Company, Tehran 14369335359, Iran; erfanpakatchi@gmail.com (E.P.);; 2 Department of Infectious and Tropical Diseases, Iranian Research Center for HIV/AIDS (IRCHA), School of Medicine, Tehran University of Medical Sciences, Tehran 1416753955, Iran

**Keywords:** human papillomavirus, HPV vaccine, quadrivalent HPV vaccine, Gardisun, Gardasil, immunogenicity, seroconversion, non-inferiority trial, cervical cancer prevention

## Abstract

**Background/Objectives:** Human papillomavirus (HPV) infection is the leading cause of cervical cancer and is associated with several anogenital and oropharyngeal malignancies. Although licensed HPV vaccines are highly effective, access remains limited in many low- and middle-income countries due to cost, supply shortages, and implementation barriers. In this study, we evaluated the immunogenicity and safety of Gardisun, a newly developed quadrivalent prophylactic HPV vaccine, compared with Gardasil^®^. **Methods:** This Phase III randomized, double-blind, active-controlled, parallel-group non-inferiority trial enrolled 450 healthy participants stratified by sex and randomized (1:1) to receive three 0.5 mL intramuscular doses of Gardisun or Gardasil^®^ on Days 0, 60, and 180. Participants were followed through to Day 210. The primary endpoint was the geometric mean titer (GMT) of antibodies against HPV types 6, 11, 16, and 18 one month after the administration of the third dose. Non-inferiority was defined as the lower bound of the 95% confidence interval (CI) for the GMT ratio exceeding 0.67. Safety was assessed through adverse event monitoring. **Results:** Of the 450 randomized participants, 422 completed the Month 7 visit and 429 received all three doses. Both vaccines induced antibody responses and seroconversion rates for all HPV types. The primary analysis met the non-inferiority criterion for HPV-6, while prespecified sensitivity analyses supported the existence of non-inferiority across all evaluated HPV types. Most adverse events were mild and transient, with no vaccine-related serious adverse events reported. **Conclusions:** Gardisun demonstrated robust immunogenicity and a safety profile comparable to that of Gardasil^®^, supporting its potential as an accessible alternative quadrivalent HPV vaccine for broader vaccination programs in resource-limited settings.

## 1. Introduction

Human papillomavirus (HPV), a non-enveloped, double-stranded DNA virus of the Papillomaviridae family, is the most common sexually transmitted viral infection worldwide. More than 200 HPV genotypes have been identified. These are broadly categorized as low-risk types, which are associated mainly with benign lesions such as genital warts, and high-risk oncogenic types, which can cause persistent infection and malignant transformation [1,2,3].

HPVs are classified into five genera, with Alpha-HPVs encompassing a wide range of clinical manifestations, from benign warts to cancers, while Beta and Gamma HPVs are primarily associated with asymptomatic skin infections [4,5].

HPV infects basal epithelial cells through microabrasions in the cervical and anogenital epithelium. Most infections are asymptomatic and resolve spontaneously; however, persistent infection with high-risk genotypes, especially HPV-16 and HPV-18, is the necessary causal factor for most cervical cancers and contributes to anal, vulvar, vaginal, penile, and oropharyngeal cancers [2,6].

HPV first binds to the basement membrane through heparan sulfate proteoglycans (HSPGs), which is then followed by engagement with secondary receptors such as α6-integrins or growth factor receptors to facilitate endocytosis [5].

High-risk HPV types drive cancer development largely through their E6 and E7 oncoproteins, which are expressed continuously once the viral genome integrates into the host DNA [5,7].

E6 proteins facilitate the degradation of the tumor suppressor protein p53, which leads to unrestrained cellular proliferation. Meanwhile, E7 binds to and inactivates the retinoblastoma protein (pRb), abrogating cell cycle checkpoints and inducing uncontrolled cellular proliferation [4,6,7].

The accumulation of host genetic mutations driven by this process results in progression from cervical intraepithelial neoplasia (CIN) to invasive carcinoma [6].

Cervical cancer remains the most clinically important HPV-associated malignancy. Globally, the burden remains substantial, with estimates exceeding 600,000 new cervical cancer cases and more than 340,000 deaths in 2020. The disease burden is disproportionately concentrated in settings with limited screening and vaccination coverage [3,6].

The contribution of HPV-16 alone to cervical carcinogenesis is 60.5%; however, when combined with HPV-18, the two types together are responsible for 75% of all cervical cancers. Five other HPV types with oncogenic potential (31, 33, 45, 52, 58) together contribute an additional 20% to the overall burden of cervical cancer [2,7].

Current prophylactic HPV vaccines are based on virus-like particles (VLPs) composed of the L1 major capsid protein. These VLPs do not contain viral genomes and are non-infectious and non-oncogenic. It self-assembles into non-infectious virus-like particles (VLPs) that closely mimic the structure of native virions [8]. Vaccination induces type-specific neutralizing antibodies that prevent the entry of HPV at epithelial microabrasion sites and, therefore, block infection of basal epithelial cells [6,9].

Licensed prophylactic HPV vaccines include bivalent vaccines targeting HPV-16 and HPV-18; quadrivalent vaccines targeting HPV-6, HPV-11, HPV-16, and HPV-18 [3,10] with protection demonstrated for up to 14 years post-vaccination in women and men aged 27–45 years [11]; and nine-valent vaccines targeting HPV-6, HPV-11, HPV-16, HPV-18, HPV-31, HPV-33, HPV-45, HPV-52, and HPV-58. Despite the availability of effective vaccines, global coverage remains far below the World Health Organization target of vaccinating 90% of girls by age 15 by 2030 [12].

Regional differences in HPV prevalence and vaccine access remain pronounced. HPV vaccination programs in countries such as Australia and the United Kingdom have shown considerable reductions in HPV prevalence. In contrast, HPV burdens have been reported in parts of Africa, Eastern Europe, Latin America, and the Middle East [13,14].

In Iran, cervical cancer remains an important preventable malignancy. More than 33 million girls and women older than 15 years are considered at risk. National and regional data indicate clinically relevant circulation of both low-risk and high-risk HPV genotypes, including HPV-6, HPV-11, HPV-16, and HPV-52 [15,16].

In countries with female vaccination coverage ≥ 50%, HPV-16 and HPV-18 infections decreased significantly by 68% and anogenital warts decreased by 61% in girls aged 13–19 years, with significant herd effects observed in unvaccinated men and women [17].

But in many low- and middle-income countries, the high cost and limited availability of imported HPV vaccines create a persistent barrier to broad immunization coverage [9].

Developing regional manufacturing capacity is considered a key strategy to improve supply stability and facilitate program implementation in areas most affected by disease [3].

A locally manufactured quadrivalent vaccine with comparable immunogenicity and safety could, therefore, have substantial public health value by improving affordability, supply resilience, and programmatic feasibility.

Gardisun is a recombinant quadrivalent HPV vaccine containing L1 VLP antigens for HPV types 6, 11, 16, and 18. The present Phase III trial was designed to evaluate whether Gardisun is immunologically non-inferior to the reference quadrivalent HPV vaccine Gardasil^®^ and to compare seroconversion and safety outcomes between the two vaccines in healthy volunteers aged 15–35 years.

## 2. Materials and Methods

### 2.1. Study Design and Oversight

This was a Phase III, randomized, double-blind, active-controlled, parallel-group non-inferiority trial comparing Gardisun with Gardasil^®^. This study was designed to assess immunogenicity and safety after a three-dose schedule administered at 0, 2, and 6 months. The protocol was developed in accordance with the principles of the International Council for Harmonisation Good Clinical Practice (ICH-GCP), applicable national regulatory requirements, and relevant WHO technical guidance for HPV vaccine evaluation [18].

The protocol was reviewed by the regulatory authority, the Data and Safety Monitoring Board (DSMB), and the contracted clinical research organization (CRO), and this study was approved by the relevant national regulatory authority and the institutional ethics committee. Study conduct, participant care, clinical assessments, and laboratory evaluations were supervised by the principal investigator team. Participant screening and recruitment were performed by resident physicians at Tehran General Hospital, Iran, and antibody titer analyses were performed at Noor Laboratory.

Data collection was conducted using paper case report forms (CRFs) by nursing staff and study physicians after written informed consent had been obtained from all participants and, where applicable, from their legal guardians. All data were subsequently transcribed into a validated electronic data capture (EDC) system by trained study personnel. Data management and statistical analyses were then performed under blinded conditions by the data management team and CRO representatives. The EDC workflow, implemented in accordance with Good Clinical Data Management Practice (GCDMP), included predefined validation checks, discrepancy management, and audit trails to ensure data accuracy, integrity, and protocol compliance throughout the study.

### 2.2. Participants

**Figure 1:**
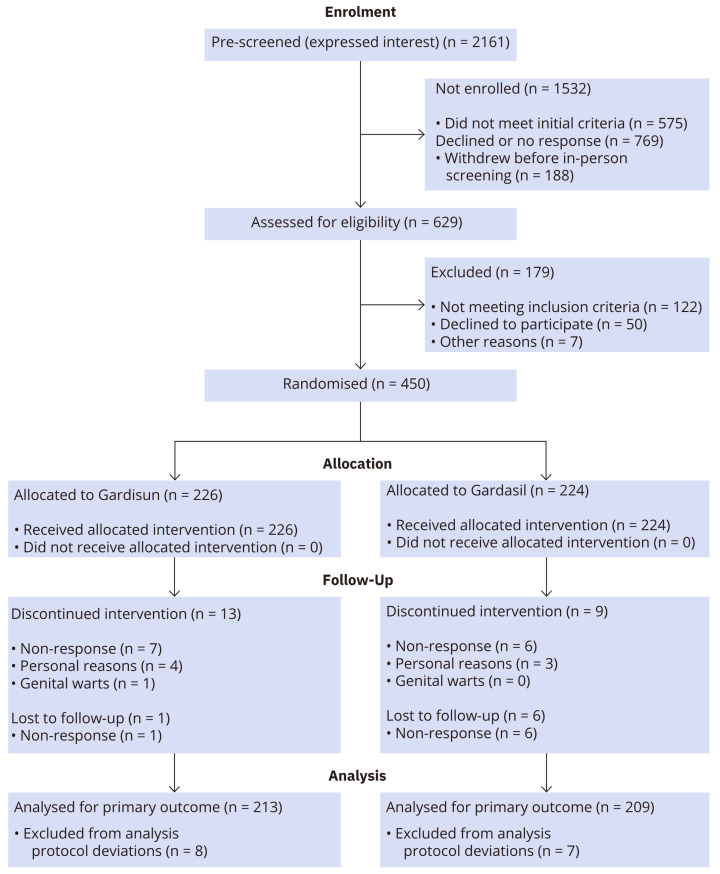
Participant flow throughout this study. Enrollment age: 15–35 years.

Healthy male and female volunteers aged 15–35 years were recruited from an initial pool of approximately 2200 interested individuals, as shown in Figure 1. Screening included medical history, physical examination, laboratory testing, and dermatologic and anogenital assessment to exclude suspicious HPV-related lesions, and baseline serologic testing for HPV types 6, 11, 16, and 18. Eligible participants were required to be in good general health; have acceptable baseline laboratory parameters, including aspartate aminotransferase ≤ 45 IU/L and alanine aminotransferase ≤ 60 IU/L; and be seronegative for HPV types 6, 11, 16, and 18 at baseline. Female participants were required to undergo a negative beta-human chorionic gonadotropin test and to agree to effective contraception throughout the seven-month study period. Key exclusion criteria included a previous abnormal Pap smear, clinically diagnosed genital warts, prior HPV vaccination, receipt of any vaccine within 21 days before enrollment, receipt of immunoglobulin or blood products within six months before baseline, uncontrolled chronic disease, type 1 or type 2 diabetes, chronic liver or kidney disease, heart failure, HIV infection, lupus, other clinically relevant immune disorders, or use of immunosuppressive therapy.

### 2.3. Randomization and Masking

Participants were randomized in a 1:1 ratio using a stratified block randomization scheme to ensure sex balance across treatment groups. Separate random numerical sequences were generated for male and female strata in blocks of four. The randomization sequence was generated by the randomization officer using permuted block randomization with a block size of four within each sex stratum. A randomization officer assigned each eligible participant a three-character code, which was used for vial labeling and participant tracking in the electronic case report form.

Allocation concealment was maintained using sealed, opaque envelopes containing treatment assignments at the study site. Only the randomization officer and the unblinded preparation nurse had access to treatment allocation information. Participants, investigators, injection staff, outcome assessors, laboratory analysts, and statistical analysts remained masked to treatment allocation. Because Gardisun was supplied in vials and Gardasil^®^ in prefilled syringes, an unblinded preparation nurse who was not involved in clinical assessment prepared visually identical syringes in a separate area. Labels were removed from the reference vaccine syringes, and the study vaccines were prepared in visually identical syringes with matching plungers and stoppers. Emergency unblinding envelopes were maintained but were not used during the trial. Laboratory samples were coded with unique identifiers and analyzed using treatment labels A and B only.

### 2.4. Vaccines and Procedures

Participants assigned to the test group received Gardisun, a quadrivalent HPV vaccine containing recombinant L1 VLPs for HPV types 6, 11, 16, and 18 produced in Hansenula polymorpha. Participants assigned to the control group received Gardasil^®^, the reference quadrivalent HPV vaccine. Both vaccines were administered as 0.5 mL intramuscular injections into the deltoid muscle.

Vaccinations were administered by trained study nurses according to the protocol-defined schedule. The vaccination schedule consisted of three doses administered on Day 0, Day 60 (±7 days), and Day 180 (±7 days), with an interval of at least 120 days between the second and third doses. Participants were observed at the study site for 30 min after each injection to monitor immediate reactions, including acute allergic events. Follow-up telephone contacts were conducted on Days 7 and 30 after the administration of first and second doses and on Day 7 after the administration of the third dose. A final on-site visit was conducted on Day 210, corresponding to 30 days (±7 days) after the third dose, for physical examination, dermatologic/anogenital assessment, and blood sampling for the primary immunogenicity analysis.

### 2.5. Vaccine Characteristics

Table 1 compares Gardasil with other quadrivalent HPV vaccines approved by major regulatory agencies, highlighting key compositional differences (recombinant expression system and adjuvant). This comparison helps to clarify how these vaccines differ beyond their shared HPV type coverage.

### 2.6. Outcomes

The primary immunogenicity endpoint was the GMT of antibodies against HPV types 6, 11, 16, and 18 measured one month after the administration of the third dose (Month 7 after first vaccination).

The per-protocol (PP) population for immunogenicity analyses comprised participants who were seronegative at baseline, received all scheduled vaccine doses within the predefined protocol windows, had evaluable Month 7 antibody data, and had no major protocol deviations affecting immunogenicity evaluation.

Antibody titers were quantified using validated quantitative ELISA assays specific for HPV types 6, 11, 16, and 18, with assay validation conducted in accordance with ICH Q2(R1) guidelines and established frameworks (CLSI EP17 and Westgard principles). Non-inferiority for the primary endpoint was concluded if the lower bound of the two-sided 95% confidence interval (CI) for the GMTR (Gardisun/Gardasil) exceeded 0.67 [19].

Serostatus was defined based on assay-specific cut-off values determined via receiver operating characteristic (ROC) curve analysis in-GraphPad Prism version 8.4.3, using baseline seronegative and post-vaccination samples. For seroconversion endpoints at Month 7, non-inferiority was assessed by ensuring that the lower bound of the 95% CI for the difference between groups was greater than −10%. Safety endpoints were analyzed in the as-treated population, defined as all randomized participants who received at least one dose of the study vaccine. Analytical sensitivity was characterized according to CLSI EP17 guidelines, including the estimation of limit of blank (LOB) and limit of detection (LOD).

Seroconversion was defined as the conversion from seronegative status at baseline to antibody positivity above the prespecified assay cut-off and was assessed at Months 2, 6, and 7 among participants who were seronegative at baseline. Secondary immunogenicity endpoints included seroconversion rates at each assessment time point and the proportion of participants achieving at least a four-fold increase in antibody titers from baseline. Antibody titers were summarized as geometric mean titers (GMTs), whereas seroconversion rates and four-fold titer increases were summarized as proportions, each with corresponding 95% confidence intervals. Safety endpoints included solicited local and systemic reactions assessed during the 30-min post-vaccination observation period and throughout the 7 days following each vaccination, unsolicited adverse events (AEs) collected through 30 days after each dose, and serious adverse events (SAEs) monitored throughout the study period. All reported AEs were coded using the Medical Dictionary for Regulatory Activities (MedDRA) and classified according to System Organ Class (SOC) and Preferred Term (PT). Adverse events were further categorized by severity (mild, moderate, or severe), and their relationship to vaccination was assessed according to predefined causality categories (certain, probable/likely, possible, or not related). Safety data were summarized across predefined post-vaccination intervals.

### 2.7. Statistical Analysis

Statistical analyses were performed according to a prespecified statistical analysis plan (SAP) finalized prior to database lock and unblinding. Participants were assigned in a 1:1 ratio to the intervention groups using stratified block randomization with a block size of four to ensure balance by sex across study arms. The per-protocol population—comprising participants who were seronegative at baseline, received all three vaccine doses within protocol-defined windows, and had evaluable Month 7 immunogenicity measurements—served as the primary analysis set, while safety analyses were conducted in the as-treated population.

To satisfy the normality assumption of linear regression models, antibody titers—which typically exhibit a right-skewed distribution—were log10-transformed prior to analysis.

We targeted 200 participants per group to achieve 95% statistical power for non-inferiority testing of Gardisun versus Gardasil^®^ (one-sided α = 0.05). Assuming a standard deviation of 1.2 and a non-inferiority margin of 0.67, this was inflated to 450 total participants to allow for a 10% attrition rate.

Primary geometric mean titer ratios (GMTRs) and corresponding one-sided 95% confidence intervals (CIs) were estimated using multivariable linear regression models with treatment group as the main explanatory variable, adjusted for age and sex as predefined prognostic covariates. To facilitate interpretation on the original antibody scale, model estimates were exponentiated. The primary endpoint was considered non-inferior if the lower bound of the one-sided 95% CI for the GMTR exceeded 0.67, a margin defined a priori based on Iranian Food and Drug Organization guideline CT-G-19 and World Health Organization recommendations [19], corresponding to a maximum acceptable 1.5-fold reduction in antibody response relative to the reference vaccine.

Secondary immunogenicity endpoints included seroconversion rates and the proportion of participants achieving a four-fold or greater increase in antibody titers from baseline. Seroconversion was defined as the transition from seronegative status at baseline to antibody concentrations exceeding genotype-specific assay cut-offs. These cut-offs were established using receiver operating characteristic (ROC) curve analysis to optimize sensitivity and specificity based on baseline seronegative and Month 7 post-vaccination samples. For seroconversion, non-inferiority was defined as a lower bound of the 95% CI for the between-group difference greater than −10%.

Sensitivity analyses were prespecified to evaluate the robustness of the immunogenicity results. The outlier-handling strategy was defined while the study remained blinded. Following review of accumulating data patterns during a Data and Safety Monitoring Board (DSMB) meeting, sensitivity analyses were conducted using two approaches: an interquartile range (IQR)-based method, in which observations below Q1 − 1.5 × IQR or above Q3 + 1.5 × IQR were excluded, and a trimmed-mean approach excluding extreme values from both tails of the antibody titer distributions. Across both analytical approaches, the lower bounds of the 95% confidence intervals for the GMTRs remained above the predefined non-inferiority margin for all four HPV types, supporting the consistency of the immunogenicity results.

The DSMB convened at four prespecified time points, including one emergency session and an interim safety review after 50% of participants had completed the three-dose schedule. Throughout the trial, the board monitored safety via serious adverse event (SAE) reports in CIOMS format and evaluated data integrity using management reports that documented system queries, annotations, and resolution logs. The DSMB also provided operational guidance, including a recommendation to maintain a minimum 120-day interval between the second and third doses to optimize immunogenicity. At study completion, the full immunogenicity dataset (GMTR, seroconversion, and antibody response analyses) was presented to the board as part of the final review, which confirmed non-inferiority.

No item-level missing data were observed for the primary endpoint, and no imputation procedures were applied.

## 3. Results

### 3.1. Participant Disposition

Of 2161 individuals who expressed interest in participation, 629 were assessed for eligibility and 450 were randomized. A total of 226 participants were assigned to the Gardisun group and 224 to the Gardasil^®^ group Table 2. The enrollment period lasted 167 days, with the first dose administered over a 71-day window. The first doses were administered starting in July 2023, and follow-up visits and assessments continued through May 2024. Follow-up occurred one week after each dose, 30 days after the first dose, and at Month 7. An unblinded preparation nurse filled identical syringes in a separate room to maintain blinding. A separate injection nurse administered the vaccine and remained unaware of the vaccine type. Resident physicians performed medical examinations and recorded data, confirmed by the principal investigator. A separate nurse monitored participants and recorded adverse events post-injection to ensure that the outcome assessor remained blinded. Participants reported all medications used before and during this study. A total of 2099 concomitant medication cases were recorded: 952 (45.4%) in the Gardasil^®^ group and 1147 (54.6%) in the Gardisun™ group. The most frequent medications included acetaminophen, propranolol, spironolactone, sertraline, and fluoxetine. Overall, 436 participants received the second dose, 429 received the third dose, and 422 attended the Month 7 final analysis visit. Among screened participants, 30 individuals (6.7%) were identified as seropositive via quantitative ROC-based analysis and were excluded from the per-protocol immunogenicity set. Additional exclusions were due to protocol deviations, including one participant who mistakenly received a dose intended for her sister, eight participants with visit dates outside allowed windows, and six participants excluded because of positive HPV findings or genital warts. Overall, 28 participants did not complete this study: 13 in the Gardisun group and 15 in the Gardasil^®^ group. Reasons for non-completion included non-response within the protocol window (*n* = 17), personal circumstances (*n* = 10), and genital warts (*n* = 1). The final per-protocol dataset comprised 400 participants for HPV-6, 404 for HPV-11, 396 for HPV-16, and 398 for HPV-18. The trial reached its planned conclusion; it was not stopped prematurely. A standard Study Close-Out Visit was conducted. High adherence (95.3%) reflected successful follow-up strategies and volunteer retention.

### 3.2. Baseline Characteristics

As shown in Table 3 baseline demographic, anthropometric, and clinical characteristics were balanced between the two groups, with no clinically meaningful differences in sex distribution, age, weight, height, BMI, or vital signs.

### 3.3. Adherence and Protocol Compliance

Adherence to the three-dose schedule was high: 429 of 450 participants (95.3%) received all three intended doses. All participants maintained the prespecified minimum interval of four weeks between the first and second doses, and most participants maintained an interval of at least 120 days between the second and third doses.

Protocol-window deviations, including eight participants whose third dose or final visit occurred outside the prespecified window, were handled via exclusion from the per-protocol immunogenicity analysis. Adherence did not differ meaningfully between treatment groups.

### 3.4. Immunogenicity

One month after the third dose, both vaccines elicited strong antibody responses against HPV types 6, 11, 16, and 18. GMTs were numerically higher in the Gardasil^®^ group for all four types. The Gardisun/Gardasil^®^ GMTRs were 0.81 for HPV-6, 0.76 for HPV-11, 0.76 for HPV-16, and 0.80 for HPV-18.

In the primary analysis, the lower bound of the 95% CI exceeded the prespecified non-inferiority margin of 0.67 for HPV-6. The corresponding lower bounds for HPV-11, HPV-16, and HPV-18 were marginally below 0.67. Because GMT-based analyses can be sensitive to extreme observations, prespecified sensitivity analyses using the interquartile range method and truncated-mean approach were performed; these analyses supported the existence of non-inferiority across all four HPV types.

Prespecified sensitivity analyses, all of which were defined in the statistical analysis plan prior to database lock and unblinding while the study remained blinded, were conducted using IQR-based outlier handling and a truncated-mean approach to assess the stability of GMTR estimates under alternative distributional assumptions. In the IQR-based approach, values outside interquartile range-defined limits were excluded prior to the re-estimation of immunogenicity measures, whereas in the truncated-mean approach, antibody titer distributions were re-analyzed after the exclusion of extreme values at both tails. Across both analytical approaches, the lower bounds of the 95% confidence intervals for GMTRs exceeded the predefined non-inferiority margin for all four HPV types.

Seroconversion rates were high in both groups by Month 7. For HPV-6, HPV-11, and HPV-16, seroconversion approached 95–99% in both arms. HPV-18 showed slightly lower seroconversion than the other types, particularly in earlier follow-up, but rates increased substantially by Month 7. The lower bounds of differences in seroconversion rates remained within the prespecified non-inferiority framework A summary of these immunogenicity results is presented in Table 4.

### 3.5. Subgroup Analyses

Preplanned subgroup analyses assessed consistency across age groups (15–20 years versus 21–35 years), biological sex, and baseline serologic measures. Baseline demographic and vital-sign profiles were well balanced across groups, reducing the likelihood that measured immunogenicity differences were attributable to baseline imbalance. Multivariable linear regression analyses did not identify statistically significant interactions between treatment assignment and key prognostic covariates, including age and sex (interaction *p* values > 0.10). These findings support broadly consistent immune responses across the predefined subgroups.

### 3.6. Safety

The safety analysis included all 450 randomized participants who received at least one dose. During the full follow-up period, 339 participants (75.5%) reported at least one adverse event, and 1386 adverse events were recorded. At least one adverse event was reported by 174 of 226 participants (77.0%) in the Gardisun group and 166 of 224 participants (74.1%) in the Gardasil^®^ group. Most adverse events were mild and transient. Mild events accounted for 91.6% of reactions in the Gardisun group and 87.8% in the Gardasil^®^ group. Injection-site pain was the most common reaction and was reported more frequently in the Gardisun group. Headache, rhinitis, pyrexia, myalgia, fatigue, and nausea were the most commonly reported systemic events. Overall, the total number of adverse events was slightly higher with Gardisun (737 events) than with Gardasil^®^ (649 events), mainly because of injection-site pain presented in Table 5.

A total of 17 serious adverse events (SAEs) were reported during the study period, including 11 events in the Gardisun group and 6 events in the Gardasil^®^ group. The incidence of SAEs was comparable between the two groups, with no apparent imbalance. The SAEs comprised a range of clinical conditions including infectious diseases (e.g., Epstein–Barr virus infection, pharyngitis, peritonsillar abscess), trauma-related injuries (e.g., ankle fracture and shoulder dislocation), gastrointestinal and surgical conditions (e.g., appendicitis and elective surgeries such as rhinoplasty, gastric sleeve surgery, and breast prosthesis surgery), neurological/cardiovascular symptoms (e.g., dizziness and chest pain attributed to anxiety), and pregnancy-related outcomes (spontaneous or induced abortion). The onset of SAEs ranged from a few hours after vaccination (e.g., dizziness following the second dose) to approximately six months post-vaccination (e.g., peritonsillar abscess and pregnancy-related outcomes). Most events occurred within days to weeks after vaccination, with no temporal clustering observed.

All SAEs were assessed for causality by the investigators using predefined CIOMS criteria. All events were classified as unrelated to vaccination based on clinical evaluation and the presence of alternative etiologies.

## 4. Discussion

This Phase III randomized, double-blind, active-controlled trial provides comparative immunogenicity and safety data for Gardisun, a locally manufactured quadrivalent HPV vaccine, versus Gardasil^®^ in healthy volunteers aged 15–35 years.

Both vaccines induced antibody responses against HPV types 6, 11, 16, and 18 and achieved high seroconversion rates after the three-dose series. While the primary analysis demonstrated non-inferiority only for HPV-6, sensitivity analyses conducted according to the study-defined analytical approach supported non-inferiority for HPV-6, HPV-11, HPV-16, and HPV-18. These findings are consistent with previous quadrivalent HPV vaccine studies, where seroconversion rates for HPV types 6, 11, 16, and 18 were reported to be above 99% after completion of the standard three-dose schedule [10,20,21].

Sensitivity analyses using prespecified outlier-handling methods supported non-inferiority for all four HPV types. Taken together with the high seroconversion rates, the results indicate that Gardisun produces a clinically meaningful immune response, although longer-term and real-world studies are needed to confirm durability and effectiveness, although the primary immunogenicity analysis showed that Gardisun met the prespecified non-inferiority criterion for HPV-6, while the lower confidence-bound estimates for HPV-11, HPV-16, and HPV-18 were marginally below the 0.67 threshold in the crude analysis. This finding should be interpreted transparently, because non-inferiority trials depend critically on the pre-specified margin, the analysis population, assay variability, and the influence of extreme observations. These analyses were conducted while the study remained blinded, and following application of the predefined outlier-handling approaches, including IQR-based filtering and trimmed-mean analysis, a more consistent pattern of non-inferiority was observed across all four HPV types.

According to WHO guidance, a lower confidence-bound threshold of 0.67 is generally recommended for establishing non-inferiority in HPV vaccine immunobridging studies, although a threshold of 0.5 may be accepted under certain circumstances [22]. In the present study, all lower confidence-bound estimates remained above 0.67 and sensitivity analyses consistently supported non-inferiority across all four HPV types. Together with the high seroconversion rates observed in both groups, these findings support the immunological comparability of Gardisun and Gardasil.

The long-term stability and immunogenicity of the Gardisun vaccine have been validated through 24-month stability studies. These studies based on pharmacopean methods demonstrate that the vaccine retains its physicochemical and biological properties over this period, ensuring consistent immune stimulation.

The safety findings were consistent with the expected reactogenicity profile of prophylactic HPV vaccination. Injection-site pain was the most frequent adverse event and occurred more commonly among Gardisun recipients. High rates of local reactogenicity are a well-documented feature of VLP-based vaccines; in original quadrivalent vaccine trials, injection-site pain was reported by 86% of vaccine recipients [10]. This difference may reflect formulation characteristics, injection-device differences, or administration-related factors.

Specifically, this difference in local reactogenicity may be partly explained by the higher adjuvant content in Gardisun (500 μg amorphous aluminum hydroxyphosphate sulfate), compared with Gardasil^®^ (225 μg), which is known to enhance innate immune activation and can contribute to increased injection-site pain in VLP-based HPV vaccines. Importantly, systemic adverse event patterns were broadly comparable between the two groups, most reactions were mild and self-limited, and no serious adverse event was considered vaccine-related.

The public health relevance of these findings is considerable. Imported HPV vaccines remain costly and, in some settings, difficult to procure at sufficient volume. A locally manufactured quadrivalent vaccine with comparable immunogenicity and safety could improve supply security, reduce acquisition costs, and support the broader integration of HPV vaccination into national immunization strategies. These advantages are particularly important in low- and middle-income countries where cervical cancer prevention is constrained by limited access to screening and vaccination.

While nine-valent HPV vaccines provide broader type coverage, quadrivalent vaccines remain a key public health tool as they target HPV-16 and -18, responsible for approximately 70% of cervical cancers, and HPV-6 and -11, responsible for about 90% of genital warts. High coverage with these vaccine types can also generate substantial herd immunity effects, reducing HPV circulation even among unvaccinated populations [23,24].

This study has several strengths, including randomized allocation, double-blind design, active control, sex-stratified randomization, high adherence to the three-dose schedule, and assessment of both immunogenicity and safety. The inclusion of both male and female participants aged 15–35 years provides clinically relevant adult immunogenicity and safety data.

This study also has limitations. The trial was powered for immunogenicity rather than clinical disease endpoints, and HPV vaccine efficacy must, therefore, be inferred indirectly from antibody responses and seroconversion. The primary crude analysis did not exceed the non-inferiority threshold for all HPV types, making sensitivity analyses important for the final interpretation.

The follow-up period in this study was limited to one month after administration of the third dose; therefore, evaluation of the durability of immune responses, delayed safety outcomes, or potential declines in antibody levels over time was not evaluated. In addition, this study cannot confirm the long-term durability of immunogenicity or the sustained comparability of Gardisun and Gardasil^®^. Moreover, the absence of data in the primary target age group for routine HPV vaccination programs—i.e., girls aged 9–14 years—limits the direct applicability of these findings to this population.

Future research should include longer-term follow-up to evaluate the persistence of antibody responses, post-marketing surveillance to assess rare adverse events, real-world effectiveness studies, and cost-effectiveness analyses using local epidemiological and economic data. Studies in adolescents aged 9–14 years and the evaluation of alternative dosing schedules would also strengthen the evidence base for national immunization policy.

## 5. Conclusions

Gardisun induced antibody responses against HPV types 6, 11, 16, and 18 and achieved high seroconversion rates after a three-dose schedule. Although the crude primary GMTR analysis was marginal for several HPV types, prespecified sensitivity analyses and the overall immunogenicity profile supported its non-inferiority to Gardasil^®^. The vaccine was generally well tolerated, with no vaccine-related serious adverse events and a safety profile comparable to that of the reference product.

These findings support the use of Gardisun as a potential alternative quadrivalent HPV vaccine for cervical cancer prevention and broader HPV-related disease prevention, particularly in settings where access to imported HPV vaccines is constrained. Long-term follow-up and post-marketing effectiveness data will be essential to confirm the durability of protection and inform large-scale implementation.

## Figures and Tables

**Table 1:** Comparison of quadrivalent HPV vaccines.

Feature	Gardasil^®^ (Reference)	Cervavac (India)	Gardisun/Iranian Quadrivalent HPV Vaccine
Manufacturer	Merck & Co., Inc, Rahway, NJ, USA.	Serum Institute of India Pvt. Ltd, Pune, Maharashtra, India.	Biosun Pharmed, Tehran, Iran
HPV types covered	6, 11, 16, 18	6, 11, 16, 18	6, 11, 16, 18
Recombinant expression system	Saccharomyces cerevisiae	Hansenula polymorpha	Hansenula polymorpha
L1 VLP content (HPV 6/11/16/18)	20/40/40/20 μg	20/40/40/20 μg	20/40/40/20 μg
Adjuvant	225 μg amorphous aluminum hydroxyphosphate sulfate	500 μg aluminum hydroxide	500 μg aluminum hydroxyphosphate sulfate
Targeted age range in this trial	Comparator product	Not applicable	15–35 years

**Table 2:** The disposition of participants from screening through to study completion.

Disposition Step	Number of Participants
Expressed interest	2161
Assessed for eligibility	629
Not enrolled	1532
Randomized and received first dose	450
Assigned to the Gardisun group	226
Assigned to the Gardasil^®^ group	224
Received second dose	436
Received third dose	429
Completed three-dose schedule	429
Attended Month 7 visit/final analysis visit	422

**Table 3:** The baseline characteristics of study participants.

Characteristic	Gardasil^®^ (*N* = 224)	Gardisun (*N* = 226)
Male, *n* (%)	104 (46.43%)	105 (46.46%)
Female, *n* (%)	120 (53.57%)	121 (53.57%)
Age, years; mean (range) ± SD	25.54 (16–35) ± 4.59	25.80 (16–35) ± 4.65
Weight, kg; mean (range) ± SD	74.53 (38–150) ± 16.30	72.99 (37–133) ± 17.73
Height, cm; mean (range) ± SD	172.34 (152–203) ± 10.47	172.27 (150–203) ± 4.57
BMI, kg/m^2^; mean (range) ± SD	24.95 (15.03–38.08) ± 4.17	24.40 (15.2–41.27) ± 4.57
Body temperature, °C; mean ± SD	35.955 ± 0.595	35.966 ± 0.551
Respiratory rate, breaths/min; mean ± SD	17.147 ± 1.7771	16.690 ± 1.729
Heart rate, bpm; mean ± SD	80.518 ± 9.241	80.659 ± 10.466
Systolic blood pressure, mmHg; mean ± SD	102.76 ± 9.21	101.03 ± 8.92
Diastolic blood pressure, mmHg; mean ± SD	66.25 ± 7.19	65.72 ± 7.10
Baseline HPV-6 seropositivity, *n* (%)	6 (2.68%)	4 (1.77%)
Baseline HPV-11 seropositivity, *n* (%)	3 (1.34%)	2 (0.88%)
Baseline HPV-16 seropositivity, *n* (%)	7 (3.13%)	8 (3.54%)
Baseline HPV-18 seropositivity, *n* (%)	8 (3.57%)	6 (2.65%)

**Table 4:** Comparison of post-dose 3 immunogenicity outcomes between Gardisun and Gardasil^®^.

HPV Type	N Gardisun	Gardisun GMT (95% CI)	N Gardasil^®^	Gardasil^®^ GMT (95% CI)	N GMTR	GMTR Gardisun/ Gardasil^®^ (95% CI) *	N Gardisun SC	Gardisun Seroconversion (95% CI) **	N Gardasil^®^ SC	Gardasil^®^ Seroconversion (95% CI)	Difference (95% CI) ***
HPV-6	204	15.68 (14.42, 17.04)	196	21.32 (20.18, 22.52)	400	0.81 (0.76, 0.87)	204	0.95 (0.92, 0.98)	196	0.99 (0.98, 1.00)	−0.04 (−0.07, −0.01)
HPV-11	205	18.73 (16.99, 20.64)	199	28.11 (26.67, 29.63)	404	0.76 (0.71, 0.81)	205	0.95 (0.91, 0.98)	199	0.99 (0.98, 1.00)	−0.04 (−0.09, −0.01)
HPV-16	199	8.16 (7.32, 9.10)	197	11.28 (10.34, 12.30)	396	0.76 (0.68, 0.85)	199	0.96 (0.94, 0.99)	197	0.99 (0.98, 1.00)	−0.03 (−0.06, 0.00)
HPV-18	202	7.40 (6.49, 8.44)	196	10.23 (9.11, 11.39)	398	0.80 (0.70, 0.91)	202	0.91 (0.86, 0.95)	196	0.95 (0.92, 0.98)	−0.04 (−0.09, 0.01)

* Non-inferiority was concluded when the lower bound of the two-sided 95% confidence interval for the GMTR exceeded the prespecified non-inferiority margin of 0.67. ** Seroconversion rates reflect the proportion of participants achieving antibody levels above the prespecified protective threshold. *** The difference column presents absolute differences between Gardisun and Gardasil^®^; negative values denote higher responses in the Gardasil^®^ group.

**Table 5:** Adverse event profile following Gardisun and Gardasil^®^ vaccination.

Adverse Event Category	Gardisun (*N* = 226), *n* (%)	Gardasil^®^ (*N* = 224), *n* (%)
Any adverse event (at least once)	174 (77.0%)	166 (74.10%)
Injection-site pain	137 (60.61%)	108 (48.21%)
Mild	135 (59.73%)	108 (48.21%)
Moderate	2 (0.88%)	0 (0.0%)
Severe	0 (0.0%)	0 (0.0%)
Injection-site swelling	3 (1.3%)	4 (1.78%)
Mild	3 (1.3%)	4 (1.78%)
Moderate	0 (0.0%)	0 (0.0%)
Severe	0 (0.0%)	0 (0.0%)
Injection-site erythema	0 (0.0%)	4 (1.78%)
Mild	0 (0.0%)	4 (1.78%)
Moderate	0 (0.0%)	0 (0.0%)
Severe	0 (0.0%)	0 (0.0%)
Injection-site pruritus	4 (1.77%)	3 (1.34%)
Mild	4 (1.77%)	3 (1.34%)
Moderate	0 (0.0%)	0 (0.0%)
Severe	0 (0.0%)	0 (0.0%)
Headache	64 (28.31%)	51 (22.77%)
Mild	60 (26.54%)	45 (20.09%)
Moderate	4 (1.77%)	6 (2.68%)
Severe	0 (0.0%)	0 (0.0%)
Rhinitis	50 (22.12%)	55 (24.55%)
Mild	50 (22.12%)	52 (23.21%)
Moderate	0 (0.0%)	3 (1.34%)
Severe	0 (0.0%)	0 (0.0%)
Fatigue	6 (2.65%)	5 (2.23%)
Mild	6 (2.65%)	4 (1.78%)
Moderate	0 (0.0%)	1 (0.45%)
Severe	0 (0.0%)	0 (0.0%)
Pyrexia	41 (18.14%)	29 (12.94%)
Mild	40 (17.70%)	25 (11.16%)
Moderate	1 (0.44%)	4 (1.78%)
Severe	0 (0.0%)	0 (0.0%)
Myalgia	42 (18.58%)	39 (17.41%)
Mild	39 (17.25%)	34 (15.18%)
Moderate	3 (1.33%)	5 (2.33%)
Severe	0 (0.0%)	0 (0.0%)
Nausea	13 (5.75%)	16 (7.14%)
Mild	12 (5.31%)	15 (6.69%)
Moderate	1 (0.44%)	1 (0.45%)
Severe	0 (0.0%)	0 (0.0%)
Serious adverse events	11 (4.67%)	6 (2.68%)
Vaccine-related serious adverse events	0 (0.0%)	0 (0.0%)

## Data Availability

The datasets generated and analyzed during this study are available from the corresponding author upon reasonable request, subject to regulatory and ethical restrictions.
